# Barriers, enablers, benefits, and drawbacks to point-of-care testing: a survey of the general practice out-of-hours service in Scotland

**DOI:** 10.3399/BJGPO.2023.0094

**Published:** 2024-04-03

**Authors:** Sarah EE Mills, SM Babar Akbar, Virginia Hernandez-Santiago

**Affiliations:** 1 University of St Andrews, School of Medicine, North Haugh, St Andrews, UK; 2 NHS Fife, General Practice Out of Hours Department, Victoria Hospital Kirkcaldy, Kirkcaldy, USA

**Keywords:** point-of-care testing, general practice out of hours, Scotland, primary health care

## Abstract

**Background:**

The general practice out-of-hours (GPOOH) service is under pressure to treat more patients in less time, while reducing referrals and minimising diagnostic errors. Point-of-care (POC) testing involves rapid clinical tests that can be used to generate results during the consultation, and has the potential to facilitate managing these competing demands safely.

**Aim:**

To describe current availability of POC tests in GPOOH in Scotland, and identify barriers, enablers, benefits, and drawbacks to its use.

**Design & setting:**

Cross-sectional mixed-methods study, which surveyed opinions of clinicians working in the GPOOH service in NHS Scotland.

**Method:**

An electronic questionnaire was developed, designed, piloted, and distributed to clinicians, which had closed questions and areas for free text.

**Results:**

In total, 142 responses were received. Urine dipstick testing (99.2%), pregnancy tests (98.5%), oxygen saturation (97.7%), and blood glucose testing (93.9%), were the only POC tests commonly available in GPOOH in NHS Scotland. There was strongest support for the provision of POC tests, particularly C-reactive protein (CRP; 79.4%), strep A (76.0%), and D-dimer (75.2%). Responders felt that POC tests would improve confidence (92.3%) and safety (89.8%) surrounding clinical decision making, improve patient satisfaction (80.6%), and reduce hospital and secondary care referrals (77.5%). Barriers to POC test use were availability of the test kits and machines (94.5%), training requirements on how to use the machine (71.1%) and interpret results (56.3%), and time to do the test (62.0%).

**Conclusion:**

Few POC tests are in regular use in GPOOH in Scotland. GPOOH clinicians are supportive of using POC testing. They identified a number of benefits to its use, with very few drawbacks. Increased provision of POC testing in GPOOH in NHS Scotland should be considered urgently.

## How this fits in

Point-of-care (POC) testing is routinely used in emergency and urgent care in many countries and across multiple settings; however, its use in the general practice out-of-hours (GPOOH) setting in Scotland has not been evaluated. Very few studies have examined POC test use in GPOOH; those that have done so indicate that its use was incredibly variable and that clinicians encountered a number of barriers to its use including time, practical challenges, and doubt as to its utility. This research gives an overview of what POC tests are currently in use across Scotland, and identified which tests clinicians in GPOOH in NHS Scotland think would be useful. It identified potential barriers and enablers to the introduction of POC testing in GPOOH in Scotland.

## Introduction

General practice out-of-hours (GPOOH) service forms a key part of the Scottish ‘unscheduled care’ system, by providing medical care, available to the public without prior appointment or arrangement, outside of core hours.^1^ The GPOOH service is under unsustainable and increasing pressure owing to the combinations of rising demand, increasing medical complexity, and shrinking workforce.^2–8^ The past few years have seen an increase in GPOOH shift vacancies, nurses covering GP shifts, lack of triage cover, and use of standby clinicians.^3^ Consultations in GPOOH often involve high-risk decision making because patients presenting to GPOOH have a higher prevalence of acute illness, and clinicians working in GPOOH have limited access to medical information and laboratory results, without any prior knowledge of the patient or continuity of care.^8,9^

In Scotland, GPOOH is accessed through the NHS 24 system. NHS 24 is a clinical support service accessed through calling ‘111’. It is able to direct patients to the best place to address their clinical concerns, including pharmacists, dentists, in-hours GPs, accident and emergency (A&E), and GPOOH. Patients are directed to GPOOH when they have a clinical need that requires medical input, and where that input is required during times where their normal GP surgery is closed (weekends, public holidays, evenings, and overnight).^8^

Point-of-care (POC) testing involves clinical tests taken on-site at the time of consultation, with results available quickly enough to be used during the consultation; they tend to give a numerical value and replace formal laboratory testing.^10–14^ POC tests should be distinguished from POC investigations, such as electrocardiograms (ECGs) and bedside ultrasound, which are more time-consuming to deliver, and which provide non-numerical values. POC testing has the potential to improve clinical outcomes in primary care by improving prescribing accuracy, reducing unnecessary referrals, maximising effectiveness of care, and reducing costs.^9–11,15–17^ However, existing evaluations of POC testing are usually based on secondary care, rather than primary or unscheduled care, and largely focus on diagnostic accuracy rather than clinical impact.^12,15,18–24^ There is a lack of evidence of its impact on primary care pathways and outcomes.^11,15,22,25–27^ POC testing features in national guidelines for respiratory infections and can be used, at clinician discretion, in assessing a large variety of clinical conditions.^12,22,26,28^ In spite of this, there is relatively little research investigating the availability and impact of POC tests in primary care, and even less of an assessment of their use in GPOOH.^9,15,29,30^ The existing literature regarding barriers and enablers to using POC tests in primary care and GPOOH is summarised in Box 1.

Box 1 **Findings from literature review: barriers and enablers to using point-of-care tests (POCTs) in primary care and general practice out-of-hours (GPOOH)**

**Table:** 

Barriers and enablers to using POCTs in primary care and GPOOH
**Enablers to using POCTs**
Making safer clinical decisions^9,15,26,29,42,43,48,49^
Feeling more confident about clinical decision making^9,15,26,29,42–44,48,49^
Improved patient satisfaction^9,29,30,43,48^
Reduce hospital admissions or referrals to secondary care^9,26,29,43,48,49^
Helpful when getting advice from colleagues remotely or over the phone^26,29^
Improved job satisfaction for clinicians^9,26,29,44^
**Barriers to using POCTs**
Takes time to do the test^9,15,26,29,30,42,43^
Additional training needed for clinicians in how to use the machine or test^9,15,26,29,30,43,44^
Additional training needed for clinicians in how to interpret the test results^9,15,26,29,30,43,44^
Availability of the machine or test kit^9,15,29,30,43^
Difficulty in explaining the test to patients^9,15,29,43,44^
Patients may not want the test^9,15,29,43^
Unsure about how accurate the test is^9,15,26,29,30,42–44^
Concerns about breaking or losing the equipment^9,29,42,43^

GPOOH is under pressure to treat patients more rapidly than ever before, while at the same time reducing referrals and minimising diagnostic errors.^9^ POC tests have the potential to facilitate managing these competing demands as safely as possible.^9,11^

However, the provision of POC testing in GPOOH is variable and dependent on individual health boards, with no cohesive national strategy for implementation, and no record of the variability of POC testing across Scotland. This study sought to identify what the range of current practice is in the provision of POC testing in GPOOH in NHS Scotland, and to determine what GPOOH clinicians’ views are on the use of POC tests in GPOOH.

### Aims

To identify what POC tests are being used in the GPOOH service in NHS Scotland and to identify what barriers, enablers, benefits, and drawbacks exist to the use of POC tests in the GPOOH service in Scotland.

## Method

This cross-sectional study surveyed opinions of clinicians working in the GPOOH service in NHS Scotland using an electronically distributed questionnaire. A literature review was carried out to identify any potential barriers, facilitators, benefits, and drawbacks to using POC testing in a GPOOH setting, and to determine which POC tests should be included in the survey. The findings from this review were used to develop and design the questionnaire (Box 1). The questions were informed by previous research and peer reviewed by an expert advisory group for relevance and comprehensibility.

The questionnaire (Appendix S1 in the supplementary material) collected demographic information about the responders, and asked them to answer questions relating to existing provision of POC tests, and to anticipated barriers, enablers, benefits, and drawbacks of introducing POC tests in the GPOOH service. It contained questions using the literature-identified potential barriers and enablers, and free-text spaces for unprompted responses.

The questionnaire was piloted and refined ahead of distribution, including adding additional POC tests suggested by the pilot group. Each question was peer reviewed by an advisory group comprised of 10 clinicians who are clinically active across in-hours and out-of-hours general practice. Questions were reviewed for content, relevance, and comprehensibility. Feedback was incorporated into the questionnaire, which was redistributed to advisory group members before being finalised and distributed.

### Sample size

Using the Qualtrics XM sample size calculator, a target sample size of 90–301 responders was calculated as being sufficiently powered to give results with a 95±5–10% confidence interval (CI).^31^

### Population and setting

The questionnaire was distributed in February 2023, with a reminder follow-up being sent in March 2023. Results were collated in April 2023. The questionnaire was delivered electronically using the Qualtrics XM tool, and links to the questionnaire were distributed by email to clinicians working in the GPOOH service, via the clinical leads for the GPOOH service in each region. Each region distributed it to clinicians in their area; however, most health boards were unable to confirm how many clinicians were on their distribution list, making it impossible to quantify a national response rate. For illustrative purposes, the questionnaire was returned by 33.6% (*n* = 42/125) of clinicians on the NHS Fife GPOOH distribution list. In order to participate in the questionnaire, participants were asked to confirm that they were clinicians working in any GPOOH service within Scotland.

### Data analysis

The data were analysed with a mixed-methods approach. Quantitative results were analysed with descriptive statistics using SPSS (version 25). Qualitative results were analysed using thematic synthesis.

## Results

### Responders

This questionnaire generated 142 responses from clinicians working in GPOOH, making it sufficiently powered to give results with 95±8 CI% (Table 1).^3,31^ The survey responders represented 8.5% of the GPOOH workforce in Scotland, therefore exceeding other previous comparable national surveys of primary care clinicians with response rates equivalent to 0.18%–7.1% of practising clinicians.^15^ Data received through this survey were compared with national workforce data for the GPOOH service (Appendix S2 in the supplementary material).^3^

**Table 1.** Responder profile versus Scottish national GPOOH workforce profile: job titles, age, and years of GPOOH service

	Survey responders (*n* = 142) (% of responders) [% of responders per role]	GPs in Scottish GPOOH workforce^3^ (% of workforce *n* = 1672 clinicians) [% within role *n* = 1378 GPs]	Nurses in Scottish GPOOH workforce^3^ (% of workforce *n* = 1672 clinicians) [% within role *n* = 294 nurses^a^]
**Job title (*n* = 142 survey responders)**			
GP	102 (71.8) [93.6]	1378 (82.4) [100]	n/a
GP trainee	7 (4.9) [6.4]		
ANP	22 (15.5) [81.5]	n/a	185 (11.1) [63.0]
Nurse (other than ANP)	5 (3.5) [18.5]	n/a	109 (6.5) [37.0]
Healthcare assistant	3 (2.1)	^b^	^b^
Paramedic practitioners	3 (2.1)	**^b^**	^b^
**Age (*n* = 138 survey responders)**			
<35 years	19 (13.8)	275 [20.0]	43 (43.0)
35–39 years	17 (12.3)	506 [36.7]	
40–44 years	24 (17.4)		
45–49 years	29 (21.0)	344 [25.0]	34 (34.0)
50–54 years	19 (13.8)		
55–59 years	18 (13.0)	134 [9.7]	15 (15.0)
60–64 years	8 (5.8)	72 [5.2]	8 (8.0)
≥65 years	4 (2.9)	47 [3.4]	
**Number of years worked in current role (*n* = 140 survey responders)**			
<5	53 (37.9)	*No national data available*	
5–10	31 (22.1)		
10–15	17 (12.1)		
15–20	18 (12.9)		
20–25	7 (5.0)		
25–30	6 (4.3)		
>30	8 (5.7)		

^a^The Primary Care Out of Hours Workforce Survey 2022 indicates that there are 294 nurses working within GPOOH, but only received demographic information for 100 nurses.^b^Healthcare support workers and paramedics were the most commonly reported additional staff types in the GPOOH Workforce Survey, and were employed in seven of the 14 health boards. No data was recorded in terms of headcount or demographics.

ANP = advanced nurse practitioner.

Among responders, 76.8% (*n* = 109) were GPs or GP Trainees, 19.0% (*n* = 27) were nurses or advanced nurse practitioners (ANPs), and 2.1% were either healthcare assistants (*n* = 3) or paramedic practitioners (*n* = 3). The majority of responders (71.8%) were GPs. Compared with national data, ANPs were relatively overrepresented (15.5% of responders versus 4.3% of clinicians in GPOOH nationally).^3^ Most responders were aged 45–49 years, and the majority (60.0%) had worked for GPOOH for <10 years. While responders tended to be slightly older than the national average, and were more likely to have a higher qualification level (for example, ANPs compared with other nursing staff), their roles and age followed a similar distribution to national data.

**Figure 1.**
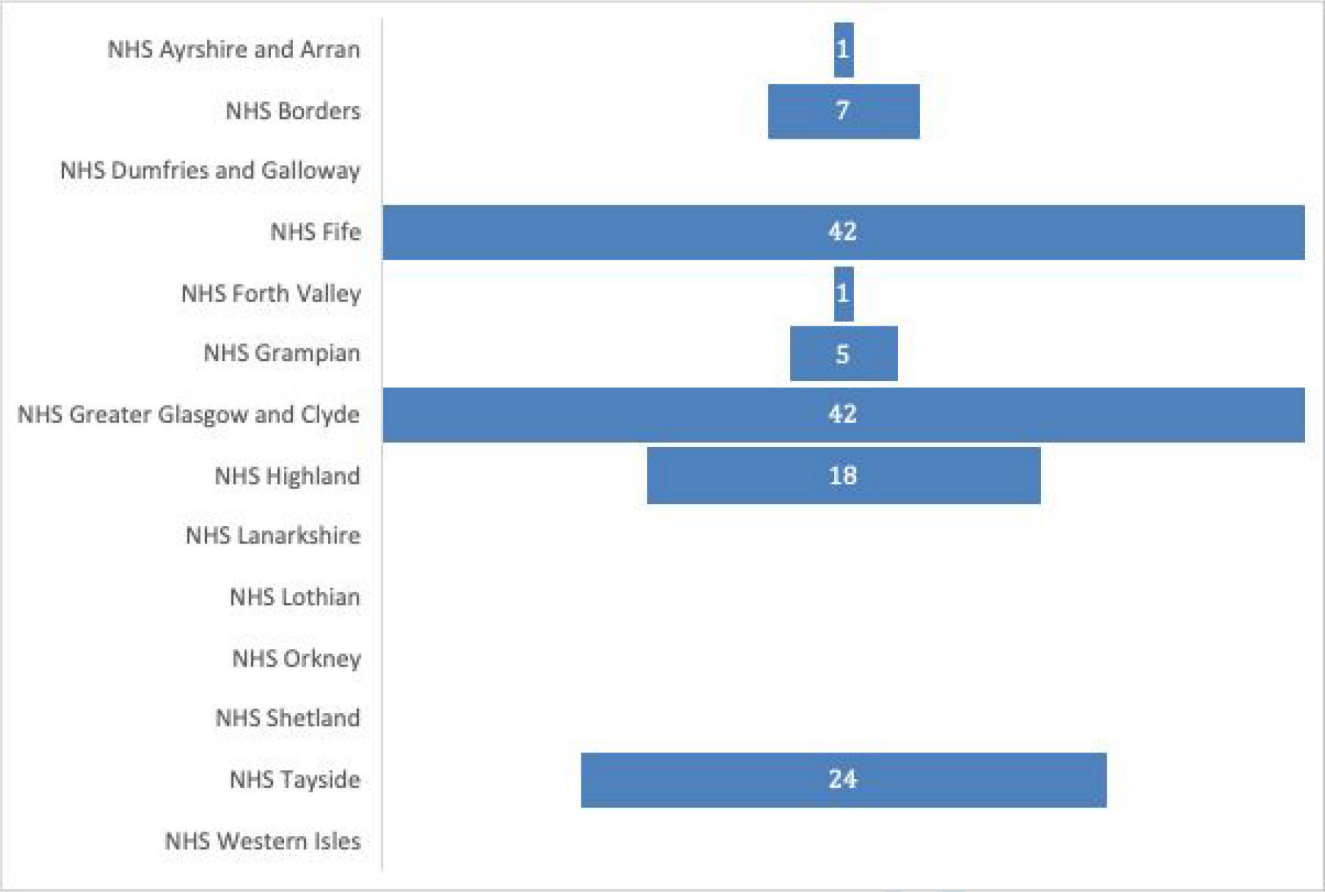
Number of questionnaire responses per health board. Missing = 2.

Responders were widely distributed across Scotland. While this survey did capture responses from 72.7% (*n* = 8/11) of Scotland’s mainland regions, there were no responses from NHS Orkney, NHS Shetland, or NHS Western Isles (Figure 1). The lack of response from the island regions may reflect that many island regions provide GPOOH cover through an ‘opt-in’ practice-level out-of-hours (OOH) service, and are not part of NHS board-run primary care OOH services.^3^ Practices-level GPOOH clinicians in these regions may not have received this questionnaire via NHS board-level distribution.

### POC testing in GPOOH: current and desired availability

**Figure 2.**
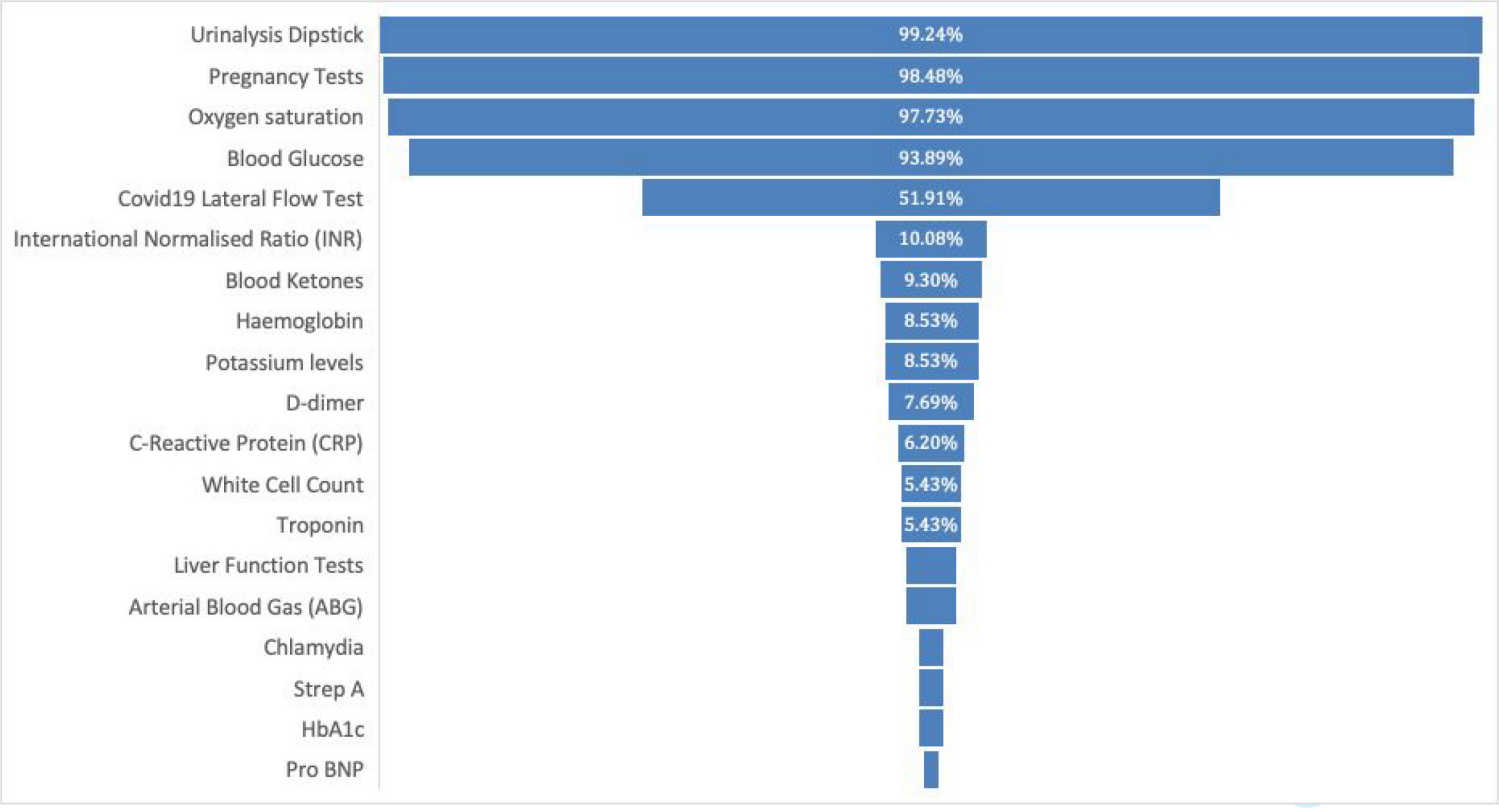
Availability of point-of-care testing facilities in your current place of work

Four POC tests — urine dipstick testing (99.2%), pregnancy tests (98.5%), oxygen saturation (97.7%), and blood glucose testing (93.9%) — were available fairly ubiquitously across GPOOH in Scotland (Figure 2). Approximately, half of GPOOH clinicians reported having access to COVID-19 lateral flow tests (LFTs). POC tests for international normalised ratio (INR), blood ketones, haemoglobin, potassium, D-dimers, C-reactive protein (CRP), white cell count (WCC), and troponins were available to 5–10% of clinicians. Liver function tests, arterial blood gases (ABGs), chlamydia, strep A, HbA1c, and N-terminal pro–B-type natriuretic peptide (NT-proBNP) tests were available to <5% of clinicians.

**Figure 3.**
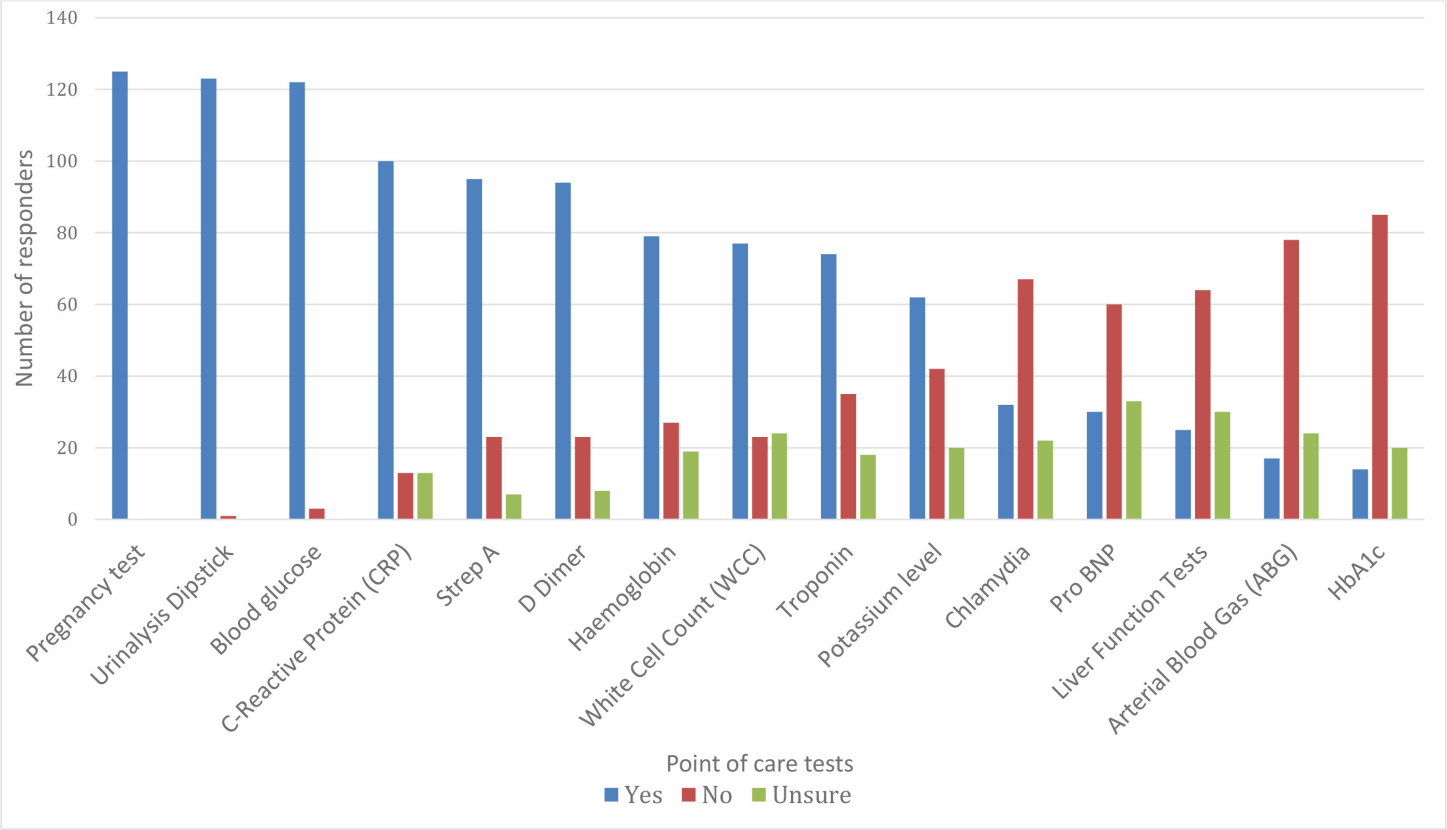
Which point of care test GP out-of-hours (OOH) clinicians would like to have available in GPOOH

In addition to the POC tests that are already widely available, there was strongest support for the provision of POC testing for CRP (79.4%), strep A (76.0%), and D-dimer (75.2%) (Figure 3). There was also significant interest in the provision of POC testing for WCC (62.1%), haemoglobin (23.2%), troponins (58.3%), and potassium (50.0%). In the free-text section, responders indicated they would also like to see POC tests available for influenza (*n* = 6), respiratory viruses (*n* = 5), renal function (estimated glomerular filtration rate; eGFR) (*n* = 4), and three-lead ECG or handheld ECG recorders (*n* = 1).

### POC testing in GPOOH: quantitative results for enablers and barriers

**Figure 4.**
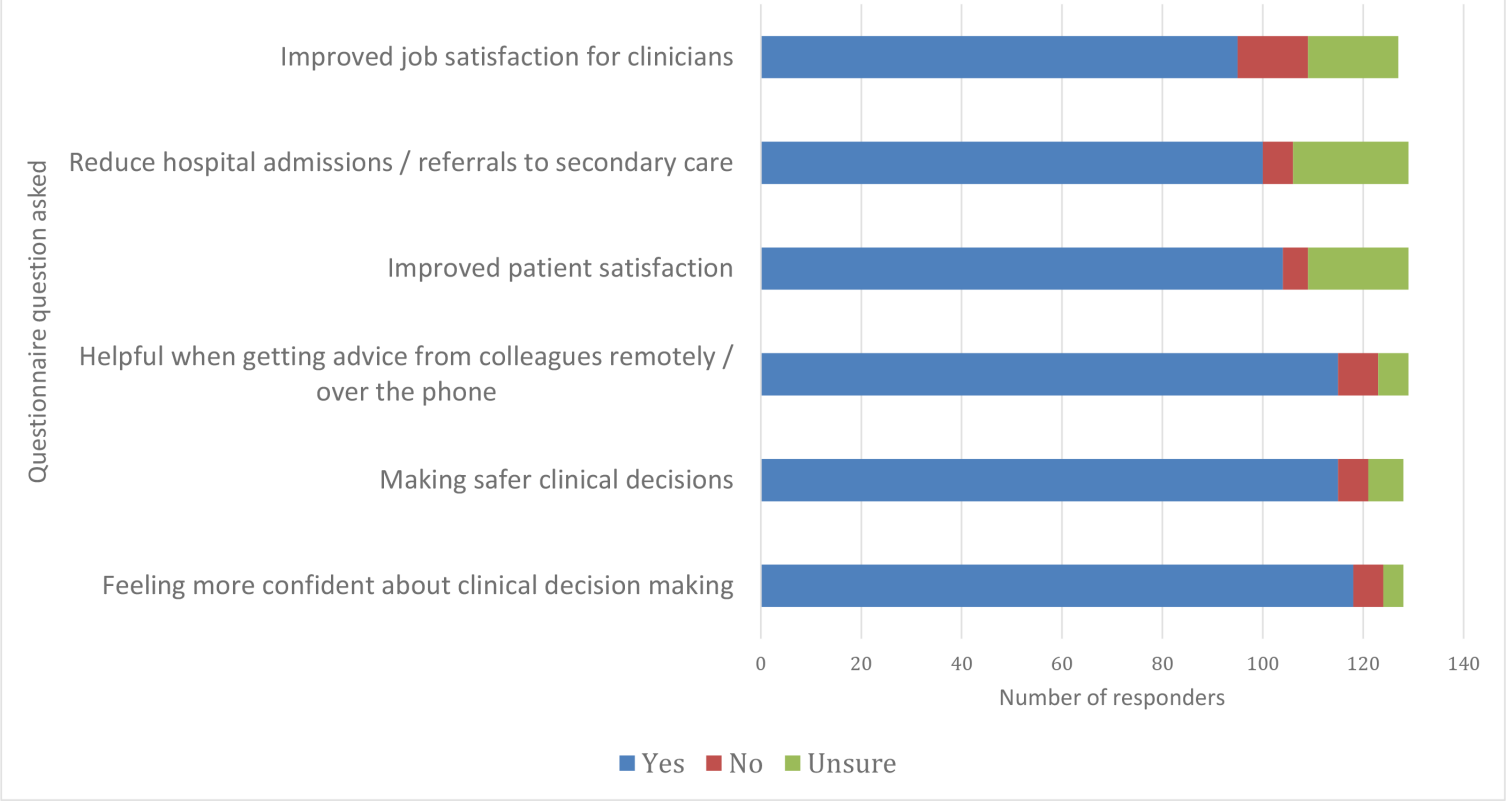
Benefits of using point-of-care testing in GP out of hours

GPOOH clinicians identified a number of benefits to the use of POC testing in GPOOH (Figure 4). Feeling more confident about clinical decision making (92.3%), making safer clinical decisions (89.8%), and finding POC testing useful when getting remote support from colleagues (89.2%) were the main benefits identified. Clinicians also felt that POC testing would confer benefits in terms of care outcomes for patients, including improve patient satisfaction (80.6%), and reduce hospital and secondary care referrals (77.5%). A substantial number of GPOOH clinicians felt that having POC tests would improve their job satisfaction (74.8%).

**Figure 5.**
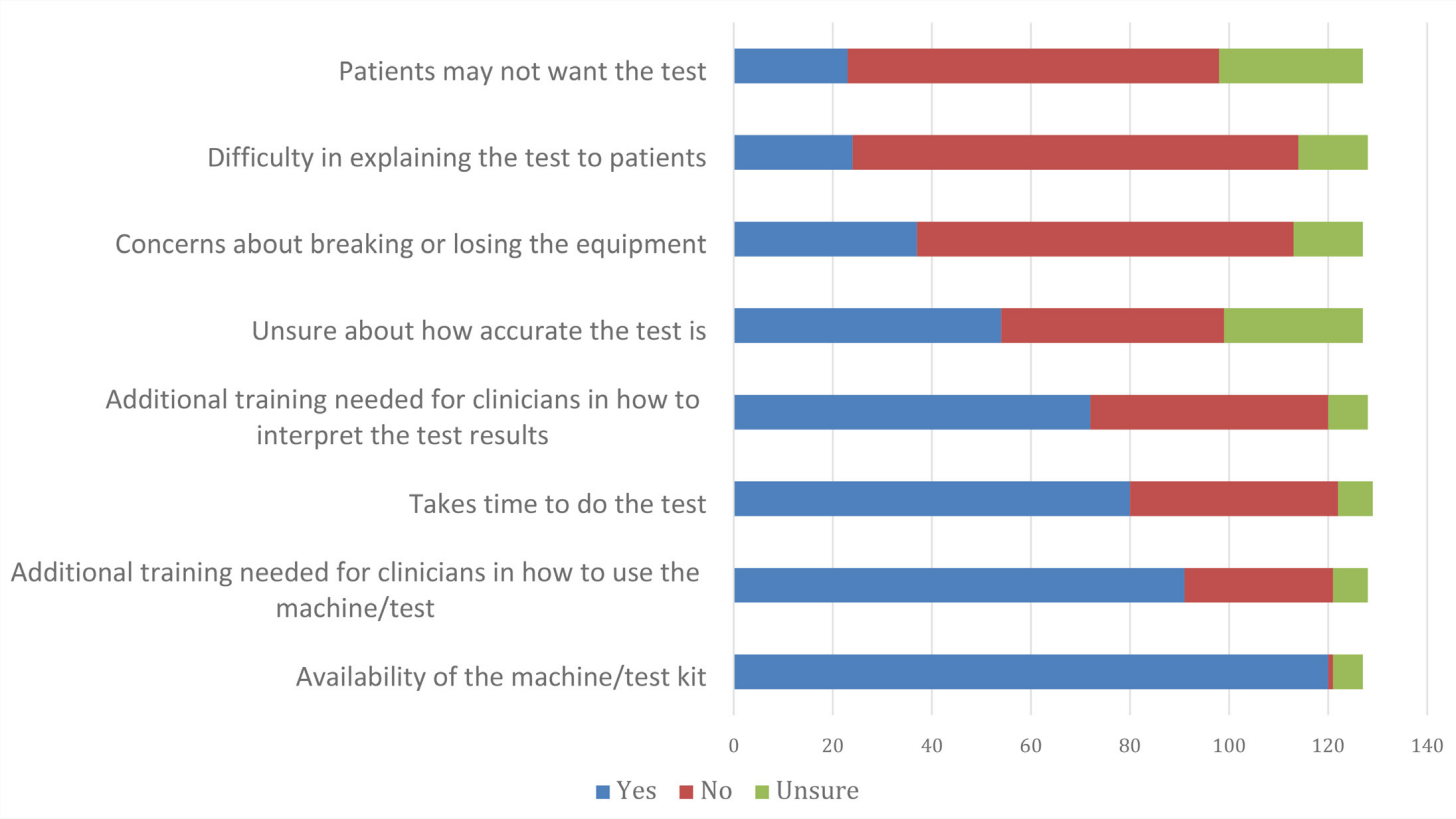
Barriers to using point-of-care testing in GP out of hours

The majority of GPOOH clinicians did not feel there would be issues with patients not wanting the test (59.1%), with difficulties explaining the test to patients (70.3%), or with concerns about breaking or losing the equipment (59.8%) (Figure 5). Barriers that were identified as being potential issues with POC testing in GPOOH in Scotland were additional training for clinicians on how to interpret results (56.3%), how to use the machine (71.1%), and the time taken to do the test (62.0%). The major barrier identified by clinicians was availability of the machine and test kit, which was identified as a barrier by (94.5%) of clinicians. There was some uncertainty regarding test accuracy, with 42.5% of clinicians saying this could be a barrier to POC test use, and 22.1% being unsure.

### POC testing in GPOOH: thematic synthesis of free text for enablers and barriers

#### Enablers

The following four interdependent themes were identified as enablers from the free-text data: time; safety; supported clinical decision making; and antimicrobial stewardship.

##### Time

GPOOH clinicians felt that using POC tests could '*reduce patient wait time for treatment*' and that it would be useful for '*targeted triage*' and appropriately assigning clinical priority to patients with POC tests indicating more severe disease.

##### Safety

GPOOH clinicians felt that POC tests would allow for '*safer discharge'* and reduce patient wait time for treatment. Some clinicians specifically commented that using POC tests would be '*medicolegally safer*'.

##### Supported clinical and shared decision making

Supported clinical decision making was a predominant theme in the free-text section. Responders commented, *'I think these tests would dramatically reduce referrals*', and that they would be *'more likely to admit patients who seem well but should be in hospital*'. One responder felt that having POC testing would make them *'more confident in ruling in or excluding diagnosis*'. Another commented that POC tests would be a '*useful aid in discussing care and treatment choices with patients*', and another that patients *'can be discharged home with safety advice with a bit more confidence*'. One responder specifically commented on the impact that this clinical decision-making support would have on training GPOOH clinicians, saying that such tests would be *'very helpful for trainees to support differential diagnosis consideration* [and] *promote teaching conversations*'.

GPOOH clinicians commented that POC tests would be a useful tool in shared decision making with patients, including '[they] *would be a useful addition to aid discussions with patients*'.

##### Antimicrobial stewardship

Responders also identified that having access to particular POC tests, including CRP and WCC, would aid in reducing unnecessary antibiotic prescribing.

### Barriers

The following five interdependent themes emerged regarding barriers to providing POC testing in GPOOH: cost; appropriateness of use; work transfer from hospitals and in-hours general practice; time; and utility and reliability. Many responders highlighted multiple themes, including one who succinctly put: *'Lack of adequate resource (staffing levels) infrastructure, agreed referral pathways and lack of time to interpret in a busy shift.*'

#### Cost

The cost of providing POC testing machines and consumables was the single biggest barrier entered in the free-text section, with 10% of responders highlighting it as a major barrier. The importance of ring-fencing funding for future costs and machine maintenance was also highlighted, with a variety of responses including, *'cost, particularly ongoing cost per test*', *'cost of the machine and consumables',* and *'kit maintenance and resupply'*.

#### Appropriateness of use

Some responders expressed concern that POC tests might become an artificial barrier or impediment to GP-led decision making; for example, refusing admission for a patient unless their CRP level was high. One responder commented, *'*[this is a] *Major barrier is that GPs are experts in risk assessments — my concern is that there will be artificial barriers to admission — ie, not accepting patients/conflicting opinions*'. Responders also expressed concern that use of POC tests might be motivated by litigious concerns and that they may, potentially inappropriately, be used in ‘*defensive medicine*’ as opposed to in patients’ best interest or at clinician discretion. One responder stated there could be *'pressure to use them to practice defence medicine. Not always in best interest of patient or appropriate use of resources including clinician time*'.

#### Work transfer from hospitals and in-hours general practice

Some tests, such as D-dimers and troponins, were seen as inappropriate work transfer from secondary care, with some GPOOH clinicians feeling that they should only be resourced and provided in a hospital setting. One responder stated, *'it feels like transfer of work from secondary care*', and another that there was *'reluctance to take on additional roles or responsibilities*'. Some responders felt that offering additional tests in GPOOH, compared with in-hours primary care, may encourage patients to present to GPOOH rather than to their own GP.

#### Time

Time to take a POC test and to interpret results was a frequent concern, with multiple responder comments, including *'time-consuming in an already time-challenged and busy service*' and '*more responsibility for OOH, not enough time*'.

#### Utility and reliability

Some clinicians queried the utility of POC testing with comments including, *'It may not change decision to admit or treat depending on how the patient presents'*, and *'reliability* [is my] *main concern*'.

## Discussion

### Summary

In spite of there being a wide range of potential for POC testing, and its widespread use in other countries and other forms of urgent and unscheduled care, there were very few POC tests in regular use in the GPOOH service in Scotland. The only POC tests in regular use across Scotland were urine dipstick testing, pregnancy tests, oxygen saturation, and blood glucose testing. Among responder GPOOH clinicians, there was strong support for the provision of further POC testing in GPOOH, particularly for CRP, strep A, and D-dimer levels.

The main potential benefits to introducing more POC tests in GPOOH in NHS Scotland include improved clinician satisfaction and confidence in clinical decision making, improved patient outcomes and patient satisfaction. GPOOH clinicians also felt that increased access to POC tests would improve their job satisfaction, suggesting that there could be a benefit to improving recruitment and retention to the GPOOH service if there was additional POC testing available.

Reducing hospital and secondary care referrals was identified as a likely benefit to having more POC testing in GPOOH. This has significant implications for the economic arguments in favour of providing POC testing to GPOOH, and warrants further investigation.

Reducing unnecessary antibiotic prescribing was identified as a potential benefit of increased access to POC CRP testing. CRP can be used as a biomarker for whether an illness is viral or bacterial, and for the severity of disease.^32^ Antimicrobial resistance (AMR) is a significant global health threat,^33^ which is currently responsible for 4.85 million deaths per year;^33^ this is expected to double by 2050.^34^ Inappropriate prescribing of antibiotics in human medicine drives global AMR.^27,35^ Studies suggest that antimicrobial prescribing in UK primary care is excessive and often inappropriate.^27,35–39^ Antibiotic prescribing rates in GPOOH are already higher than in in-hours primary care, and have increased significantly since the advent of COVID-19.^27,28,40^ In light of the overuse of antibiotics in GPOOH, strategies for using POC tests to reduce inappropriate antibiotic prescribing in the GPOOH service, should be examined in greater detail. Given the considerable number of potential benefits for increased use of POC tests in GPOOH, future pilot work and clinical effectiveness trials should be considered as a research priority.

### Strengths and limitations

This questionnaire was disseminated to clinicians currently working in GPOOH, and would therefore not have identified the views of clinicians who are not currently working in GPOOH but who might consider working there in future. It would be useful to identify whether introducing POC testing would enable more clinicians to feel more supported in providing an unscheduled care role, and whether the provision of POC testing could be used to improve recruitment and retention of GPOOH clinicians.

This survey was limited to responders in Scotland and did not take into account other parts of the UK, or other countries. There are few studies that complete national surveys of the state of provision of POC testing in GPOOH; further work in this area to identify the current situation in other parts of the UK, and in other countries, would be useful in providing a more complete frame of reference for POC test use in GPOOH.

Owing to the lack of a centrally held GPOOH information distribution system, it was not possible to contact GPOOH clinicians directly without going through health board leads. This meant the questionnaire response rate was not calculable, and differences in how this survey was electronically distributed at health-board level were likely to have had a substantial impact on the different response rates by health boards, with some health boards not returning any responses to this survey.

The responders to this survey covered 72.7% of mainland Scottish health boards (*n* = 8/11); however, responders from the island health boards were absent. Given that the delivery of GPOOH across urban, remote, and island communities varies substantially, the lack of representation from Scotland’s most remote and rural health boards is a noteworthy omission in terms of this survey’s generalisability across Scotland.^41^

### Comparison with existing literature

When presented with closed-questions highlighting barriers identified from published literature as being relevant to primary care and/or GPOOH (Box 1), the majority of responders felt that common barriers to new device or process implementations would not prove to be impediments to the use of POC tests in GPOOH in NHS Scotland.^9,15,26,29,30,42–44^ Most responder GPOOH clinicians had no concerns regarding the difficulty of explaining the test to patients, or concerns about breaking or losing the equipment, and felt that POC testing would be well received by patients.

The ability to access POC test machines and test kits while in GPOOH, clinician training in how to use the POC tests and how to interpret results, time taken to do the tests, and the cost of providing POC testing in GPOOH were the only noteworthy barriers identified to increased POC test use in GPOOH; these barriers are consistent with the existing literature.^9,15,26,29,30,42–44^

### Implications for research and practice

The findings of the study have suggested that there is interest and enthusiasm for the wider use of POC tests in GPOOH in NHS Scotland. Increased availability of POC tests in GPOOH in Scotland could improve access to POC testing in GPOOH, which would improve clinician satisfaction and confidence in clinical decision making, as well as improving patient outcomes and patient satisfaction. Future research should assess the feasibility and impact of introducing tests that are the most strongly supported by GPOOH clinicians, including tests for CRP, strep A, and D-dimer testing.

Given the distinct clinical and organisational challenges to providing GPOOH care in rural and remote areas, including an older workforce, different composition of multidisciplinary teams, challenging geography and weather, and the provision of emergency and 24/7 care,^41,45–47^ future research specifically targeting Scotland’s island health boards is needed in order to assess if the findings from this survey are generalisable to NHS Scotland’s most remote areas.

In conclusion, in spite of the variety of POC tests available, and their regular use in other settings, few tests are in regular use in GPOOH in Scotland. Clinicians in GPOOH in NHS Scotland view POC tests as a useful resource for improving patient care and clinician job satisfaction.

There are a number of benefits to POC testing, including improved accuracy and perceived support for clinical decision making, improved patient outcomes and satisfaction, reduced hospital and secondary care referrals, improved antimicrobial stewardship, and improved job satisfaction for GPOOH clinicians. Few drawbacks for POC testing were identified by GPOOH clinicians, with the main anticipated barriers being access to POC tests, training needs in using and interpreting results, time taken to do the tests, and the costs of providing POC tests.

The findings have shown that increased provision of POC testing in GPOOH in NHS Scotland should be considered as a matter of urgency.

We anticipate that introducing more POC testing will be acceptable to clinicians in GPOOH in NHS Scotland. The barriers and enablers identified in this study should be used to inform any future introduction of POC testing at regional and national levels.

## Data

The dataset relied on in this article is available from the corresponding author on reasonable request.
